# A 2-GS/s 35.9-fJ/conv.-step Voltage–Time Hybrid Pipelined ADC with Digital Background Calibration in 28-nm CMOS

**DOI:** 10.3390/mi17040495

**Published:** 2026-04-17

**Authors:** Yuan Chang, Chenghao Zhang, Yihang Yang, Chaoyang Zhang, Maliang Liu, Dongdong Chen, Yintang Yang

**Affiliations:** 1 Faculty of Integrated Circuit, Xidian University, Xi’an 710071, China; changyuan_0522@163.com (Y.C.); chzhang@163.com (C.Z.); andrewyyh@stu.xidian.edu.cn (Y.Y.); czyzhang_0913@163.com (C.Z.); ytyang@xidian.edu.cn (Y.Y.); 2 The Shaanxi Key Laboratory of Integrated Circuits and System, Xi’an 710071, China

**Keywords:** pipeline, voltage–time hybrid, digital background calibration, inverter-based push–pull RA, CC-VTC, GRO-TDC

## Abstract

This paper presents a 2-GS/s voltage–time hybrid pipelined analog-to-digital converter (ADC) with a 14-bit digital output, implemented in a 28-nm CMOS process. To alleviate the gain–bandwidth–power trade-off in deeply scaled technologies, the proposed architecture employs a SHA-less front-end and a low-gain inverter-based push–pull RA for energy-efficient coarse quantization. The residue is then transferred to the time domain via a highly linear constant-current voltage-to-time converter (CC-VTC) and digitized by a four-channel time-interleaved gated-ring-oscillator (GRO) TDC. To recover dynamic linearity degraded by low-gain amplification and interleaving mismatches, a multiplier-less digital background calibration engine is implemented. Leveraging mean absolute value (MAV) statistics and dither-injected least-mean-squares (LMS) algorithms, it effectively compensates for inter-channel and interstage errors with minimal hardware overhead. The prototype occupies an active area of 0.16 mm^2^. At 2 GS/s, the ADC achieves a Nyquist SNDR of 63.42 dB and an SFDR of 73.71 dB, corresponding to an ENOB of 10.24 bits. Consuming 86.9 mW from a 1-V supply, it achieves a Walden FoM of 35.9 fJ/conv.-step. Measurement results from multiple chips under a wide range of operating conditions verify the robustness of the proposed ADC.

## 1. Introduction

With the rapid advancement of next-generation broadband wireless communications, high-resolution radar, and ultrafast test-and-measurement instruments, and energy-harvesting intelligent sensing systems, analog-to-digital converters (ADCs) are subject to increasingly stringent requirements regarding sampling rate, resolution, and overall energy efficiency [1,2,3,4,5,6]. Among high-speed, high-accuracy conversion architectures, pipelined ADCs have remained the prevailing solution for GS/s-class medium-to-high-resolution applications due to the inherent concurrent processing capabilities [7,8]. However, as CMOS technology scales aggressively to 28 nm and beyond, the continuous reduction in supply voltage, coupled with the severe degradation of intrinsic transistor gain, imposes fundamental limitations on conventional voltage-domain pipelined ADCs [9,10]. Under these conditions, designing a closed-loop residue amplifier (RA) capable of achieving highly accurate settling within an extremely narrow clock period entails substantial power and area penalties, thereby becoming the primary bottleneck to further escalating the sampling rate.

To alleviate the longstanding gain-bandwidth-power trade-off in conventional voltage-domain designs, transferring part of the quantization process from the voltage domain to the time domain has shown considerable potential [11,12]. In contrast to voltage signals, whose dynamic ranges are fundamentally constrained by the supply limit, time-domain signals inherently benefit from the reduced gate delays enabled by advanced process scaling, thereby supporting finer quantization resolution. Nevertheless, purely time-domain ADCs remain constrained in high-resolution applications by the nonlinearities of voltage-to-time converters (VTCs) [13,14]. To overcome these limitations, voltage–time hybrid pipelined architectures have emerged as an effective solution for achieving a favorable balance among speed, accuracy, and power efficiency. In such architectures, the front-end employs a low-resolution voltage-domain ADC for fast coarse quantization and residue generation, whereas the back-end adopts an energy-efficient time-to-digital converter (TDC) for fine quantization [15,16].

Motivated by these trends and design challenges, this work presents a 2-GS/s voltage–time hybrid pipelined ADC with a 14-bit digital output in 28-nm CMOS. Compared with the voltage–time hybrid ADCs reported in [12,15], which mainly target medium-resolution designs, this work extends the hybrid architecture to a GS/s-class high-resolution pipelined ADC through a co-designed three-stage voltage–time conversion chain. Furthermore, unlike the hybrid pipelined ADC in [16], which mainly focuses on gain-error calibration, the proposed multiplier-less digital background calibration framework simultaneously compensates for inter-channel timing, offset, and gain mismatches, as well as interstage gain errors. To achieve this, the proposed design combines a SHA-less front-end, a low-gain inverter-based push–pull residue amplifier (RA), a highly linear constant-current voltage-to-time converter (CC-VTC), and a four-channel time-interleaved gated-ring-oscillator (GRO) TDC. By jointly optimizing the analog residue-transfer path and the low-overhead digital calibration engine, the proposed architecture alleviates the gain-bandwidth-power trade-off in deeply scaled CMOS while maintaining competitive Nyquist linearity and energy efficiency.

The remainder of this paper is organized as follows: Section 2 describes the overall architecture of the designed hybrid ADC and its key analog building blocks, including the SHA-less front-end, the inverter-based residue amplifier, the highly linear VTC, and the GRO-based TDC. Section 3 details the adopted background calibration algorithms used in this design. Section 4 reports the comprehensive measurement results of the prototype chip. Finally, Section 5 concludes this paper.

## 2. Circuit of the Proposed ADC

### 2.1. Overall Architecture of the Designed ADC

**Figure 1:**
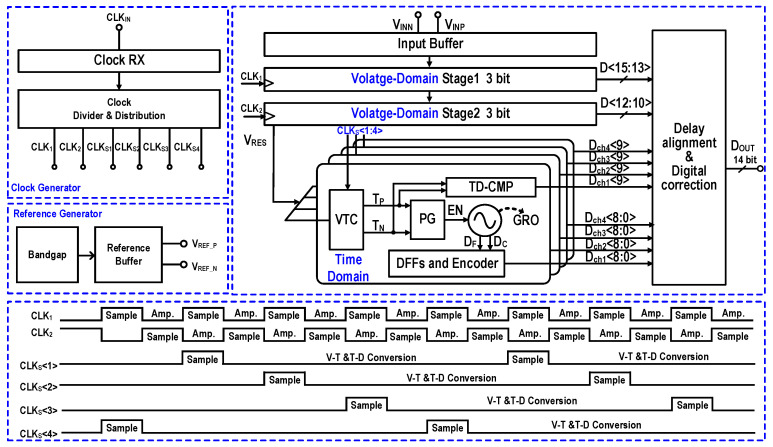
Overall architecture and timing diagram of the designed hybrid-domain pipeline ADC.

Figure 1 illustrates the top-level architecture and operating timing of the designed hybrid-domain pipelined ADC. The converter employs a three-stage pipelined architecture with 1-bit redundancy between adjacent stages to enhance tolerance against quantization errors caused by circuit non-idealities. A highly linear input buffer is integrated at the front-end of the first stage to suppress sampling kickback and support wideband input signals. The first two stages operate in the voltage domain, each utilizing a 3-bit flash ADC to achieve high-speed coarse quantization at a sampling rate of 2 GS/s. The corresponding residues are generated by capacitive DACs (CDACs), amplified by RAs, and subsequently forwarded to the following stages. To bridge the voltage and time domains, a CC-VTC is employed to convert the amplified residue voltage from the second stage into differential time-domain pulses (*T_P_* and *T_N_*) with high linearity. The third stage consists of a four-channel time-interleaved 9-bit time-domain ADC, with each channel operating at 0.5 GS/s. The GRO utilizes gating logic to quantize the VTC output pulses. By preserving its phase state during idle periods, the GRO inherently achieves first-order quantization noise shaping, while multi-phase encoders ensure high time resolution at the sub-gate delay level. Balancing the trade-offs among resolution, power, area, and bandwidth, the first-stage sampling capacitor is optimized to 1.5 pF to mitigate the penalties of excessive capacitance on wideband performance and power consumption. Benefiting from the interstage gain, the relaxed thermal noise requirements allow the sampling capacitors of the second and third stages to be scaled down to 400 fF and 25 fF, respectively.

The clocking circuitry, comprising a clock receiver, divider, and distribution network, delivers low-jitter sampling clocks and precise operational timing across all pipeline stages. An on-chip reference generator, consisting of a ban8dgap core and reference buffers, is integrated to facilitate high-speed and high-accuracy conversion. The bandgap core supplies a temperature-stable reference voltage, while the buffers ensure rapid reference settling during the conversion phases. Regarding the timing scheme, the first two stages alternate between sampling and amplification under 50% duty-cycle clocks, while the third stage utilizes 12.5% duty-cycle clocks to extend the time-domain quantization range. After synchronous on-chip processing and interstage delay alignment, the final digital outputs are driven off-chip via a high-speed data interface.

### 2.2. Clock Receiver and Jitter Analysis

To ensure high performance at high input frequencies, the sampling clock jitter must meet the stringent requirements of the ADC’s bandwidth and SNDR. The SNR limitation imposed by the total aperture jitter (*J*) can be expressed as:

(1) $$SNR=-20\log(2\pi \cdot f_{in}\cdot J)$$

where *f_in_* is the input signal frequency. At high input frequencies, this jitter-induced noise becomes one of the dominant limitations on the achievable SNDR. To maintain an SNDR equivalent to an 11-bit effective resolution at the Nyquist frequency (1 GHz), the total sampling clock jitter must be less than 42.3 fs. This jitter is accumulated from the external clock source and the internal clock distribution paths.

**Figure 2:**
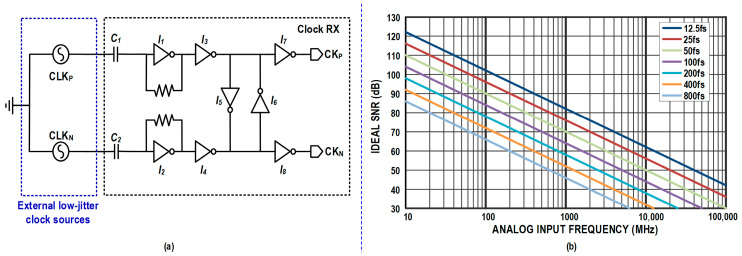
(**a**) Schematic of the inverter-based clock receiver; (**b**) Ideal SNR versus analog input frequency under different rms clock jitter levels.

Figure 2a shows the schematic of the integrated clock receiver (Clock RX). A low-power inverter-based structure is adopted to maintain compatibility with the low supply voltage in the nanoscale process. The first stage (*I*_1_, *I*_2_) utilizes AC-coupled inputs with self-biasing to amplify the external high-speed sinusoidal clock. Subsequently, *I*_3_ and *I*_4_ perform pulse shaping, while cross-coupled inverters (*I*_5_, *I*_6_) are employed to compensate for the phase mismatch between the differential clocks. Finally, *I*_7_ and *I*_8_ drive the subsequent distribution network. The size of the cross-coupled inverters is optimized to be less than one-fourth of the preceding stage to prevent jitter degradation.

Figure 2b illustrates the relationship between the ideal SNR and input frequency for various jitter levels. Post-layout simulations indicate that the designed Clock RX contributes less than 32 fs rms jitter. The bandwidth and jitter performance can be further scaled by adjusting the power consumption of the buffer chain to accommodate different application requirements. These results indicate that the proposed clocking and time-domain quantization scheme provides sufficient robustness against jitter and PVT variations for the targeted 2-GS/s operation.

### 2.3. The Designed Input Buffer

**Figure 3:**
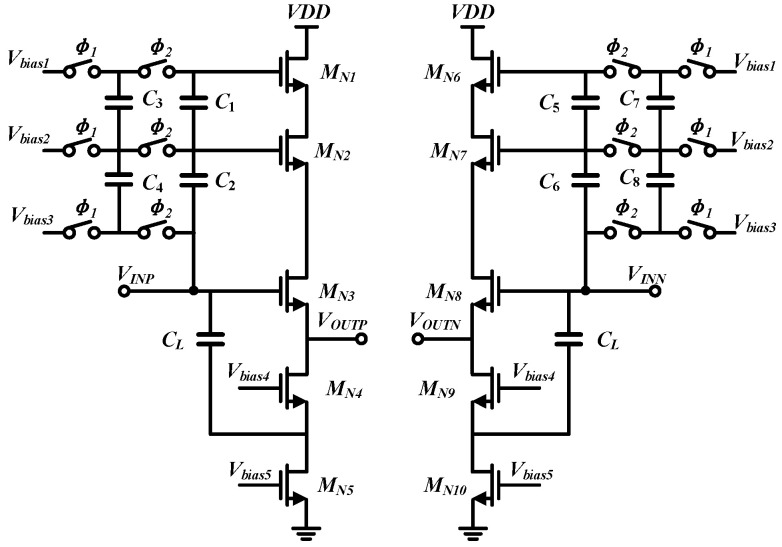
Circuit schematic of the implemented input buffer.

Figure 3 shows the circuit schematic of the designed input buffer. To suppress the severe distortion caused by the nonlinear output resistance of conventional NMOS source followers in high-speed ADC applications, this work employs an input buffer based on switched-capacitor level shifting. By combining a dual-layer bootstrap mechanism with an active feedforward capacitive path, the implemented buffer effectively improves linearity and reduces power consumption while maintaining wideband driving capability.

In this architecture, taking the left half-circuit as an example, *M_N_*_3_ acts as the main input transistor, while *M_N_*_1_ serves as a bootstrapped active load and *M_N_*_2_ functions as a bootstrapped cascode device. The dual-layer bootstrap mechanism relies on a switched-capacitor network to decouple wideband AC tracking from stable DC biasing. Specifically, capacitors *C*_1_ and *C*_2_ act as floating level shifters in the main signal path. Since the voltage across a capacitor cannot change instantaneously, *C*_2_ seamlessly couples the AC fluctuations of the input signal *V_INP_* to the gate of *M_N_*_2_, while *C*_1_ further transfers these variations to the gate of *M_N_*_1_. This dynamic tracking forces the source of *M_N_*_2_—and consequently the drain of *M_N_*_3_—to accurately follow *V_INP_*. As a result, the drain-to-source voltage *V_DS_* of the critical input transistor *M_N_*_3_ is rigidly clamped, drastically suppressing nonlinear distortion induced by channel-length modulation. To compensate for charge leakage and parasitic effects, *C*_3_ and *C*_4_ function as flying capacitors driven by non-overlapping clock phases *ϕ*_1_ and *ϕ*_2_. When *ϕ*_1_ is high, *C*_3_ and *C*_4_ are connected to the static bias voltages to sample the desired DC voltage differences. When *ϕ*_2_ is high, *C*_3_ and *C*_4_ are placed in parallel with *C*_1_ and *C*_2_, respectively, dumping the sampled charge to refresh the DC bias levels of the bootstrap nodes without interrupting the high-speed AC tracking path.

At the bottom of the source-follower branch, *M_N_*_5_ provides the foundational static bias current, while the cascode transistor *M_N_*_4_ boosts the output impedance of the current source. Furthermore, an active feedforward capacitor *C_L_*, intentionally matched to the ADC load sampling capacitance, is introduced to alleviate the high-frequency transient driving burden. Unlike conventional passive feedforward techniques, *C_L_* couples the AC component of the input directly to the source of *M_N_*_4_. Utilizing the common-gate current-buffering property of *M_N_*_4_, the transient compensation current is actively injected into the output node. This dynamic auxiliary path supplies a significant portion of the transient current required to drive the ADC sampling network, reducing the current swing of *M_N_*_3_ and maintaining a stable *V_GS_* even under low static power consumption. To improve the robustness and reproducibility of the proposed buffer, the key capacitor sizing follows specific design guidelines governed by the trade-offs among tracking accuracy, power efficiency, and layout area. First, the level-shifting capacitors (*C*_1_ and *C*_2_) are sized significantly larger than the parasitic gate-to-source capacitances (*C_gs_*) of the bootstrapped transistors (*M_N_*_1_ and *M_N_*_2_) to prevent AC-signal attenuation caused by capacitive voltage division. However, their maximum sizes are constrained by the layout area and the loading effect on the preceding driving stage. Second, the flying capacitors (*C*_3_ and *C*_4_) are utilized merely to replenish the charge loss caused by parasitic leakage and switch charge injection. To minimize dynamic switching power and suppress kT/C noise folding during the refresh phases, *C*_3_ and *C*_4_ are designed to be considerably smaller than *C*_1_ and *C*_2_, typically maintaining a relative scaling ratio of 10% to 20%. Finally, to maximize the effectiveness of the transient current compensation, the active feedforward capacitor (*C_L_*) is scaled to approximately match the equivalent single-ended sampling capacitance of the subsequent ADC network. This sizing allows *C_L_* to provide comparable transient assistance to the load and thereby reduce the dynamic burden on the main transistor *M_N_*_3_. Crucially, the reverse-isolation property of *M_N_*_4_ effectively blocks the kickback noise of the subsequent ADC stages from propagating back to the input, ensuring a cleaner front-end signal environment. Moreover, deep-N-well (DNW) NMOS devices are adopted in the main signal path to eliminate the body effect, improve substrate-noise isolation, and enhance wideband heavy-load driving capability. Post-layout simulations demonstrate that this structure achieves an approximately 10 dB improvement in linearity while reducing power consumption by as much as 70%.

**Figure 4:**
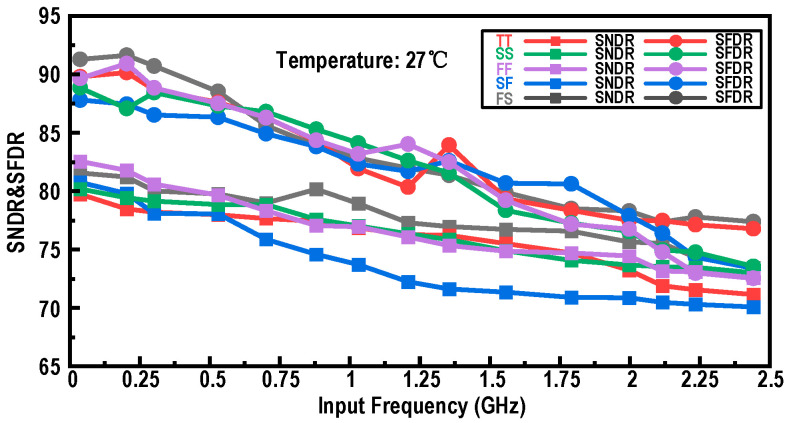
Simulated SNDR and SFDR of the designed input buffer versus input frequency across different process corners at 27 °C.

**Figure 5:**
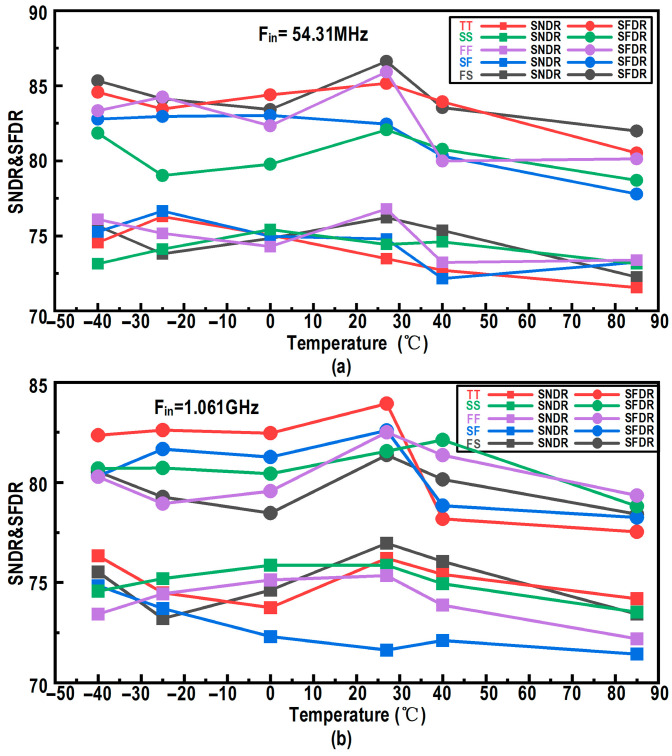
Simulated dynamic performance of the designed input buffer: SNDR and SFDR versus temperature across different process corners for (**a**) a low-frequency input (54.31 MHz) and (**b**) a near-Nyquist input frequency (1.061 GHz).

Figure 4 shows the simulated performance of the designed input buffer: as the input frequency is swept from near-DC to 2.5 GHz at room temperature, the dynamic performance indicates that the spurious-free dynamic range (SFDR) remains above 73 dB and the signal-to-noise-and-distortion ratio (SNDR) remains above 70 dB over the entire frequency range, demonstrating favorable high-frequency capabilities for high-speed, high-accuracy operation. To evaluate the circuit’s robustness against process and environmental variations, Figure 5a,b show the simulated SNDR and SFDR of the input buffer versus temperature across different process corners under low-frequency and near-Nyquist input conditions, respectively. The results confirm that, for both low-frequency and near-Nyquist inputs, the input buffer maintains an SNDR exceeding 70 dB and an SFDR consistently above 75 dB across all process corners and temperature conditions. Although this raw high-frequency SFDR differs from the ideal 14-bit mathematical limit, it fully satisfies the system-level design targets partitioned for the analog front-end. In a 2-GS/s architecture, achieving an effective raw linearity of this level provides a robust foundation for the overall ADC design. It ensures that the residual distortions, such as high-order harmonics induced by process non-idealities, remain within the correction capability of the subsequent digital background calibration, thereby supporting the intended 14-bit digital output format of the ADC. Overall, compared with a conventional input buffer without level shifting or an active feedforward capacitance, the designed structure achieves a notable improvement in SFDR, demonstrating its advantages in linearity, power efficiency, and wideband heavy-load driving capability.

### 2.4. The Designed Voltage-Domain Pipeline Architecture

**Figure 6:**
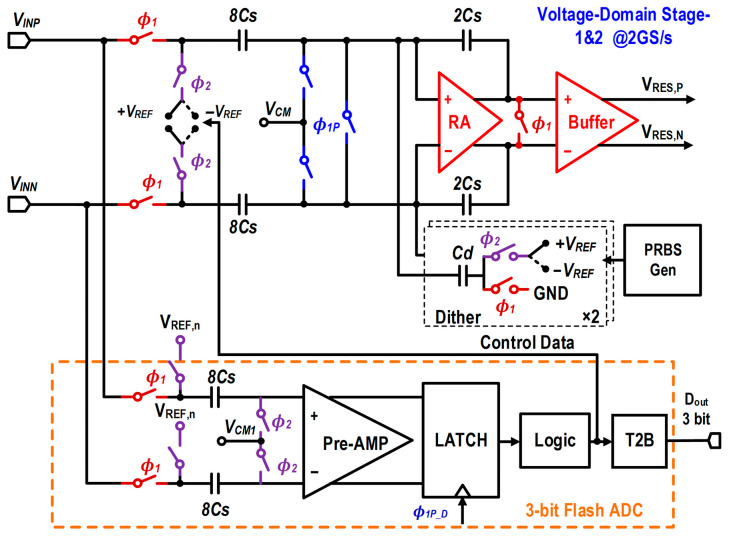
Circuit schematic of the SHA-less voltage-domain pipelined stages (Stages 1 and 2).

Figure 6 illustrates the circuit topology of the first and second stages of the pipelined ADC. To eliminate the additional noise and nonlinear distortion introduced by a conventional front-end sample-and-hold amplifier (SHA) while further reducing system power and area, the first two stages adopt a SHA-less architecture. In these stages, the sub-ADCs utilize high-speed flash structures to minimize quantization delay, thereby providing sufficient settling time for the RA within the MDAC. Furthermore, the sub-ADC incorporates a “Logic” block following the comparator latches. This block is specifically designed to perform bubble error correction on the raw comparator outputs, ensuring system reliability. Additionally, it directly generates the thermometer-coded “Control Data” to drive the DAC capacitor array within the MDAC. By bypassing the Thermometer-to-Binary (T2B) conversion for the DAC feedback path, the logic delay is reduced, which helps preserve the settling time at the 2 GS/s operating speed. Concurrently, the corrected outputs are passed to the T2B block to generate the final 3-bit digital output (D_out_).

**Figure 7:**
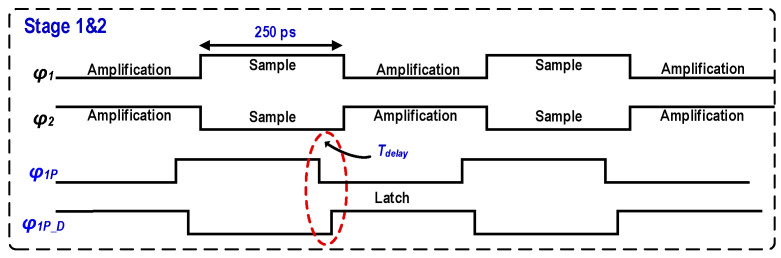
Timing diagram of the first two pipelined stages.

The timing relationship of the first two stages is shown in Figure 7. Both stages are driven by two-phase non-overlapping clocks (*ϕ*_1_ and *ϕ*_2_) with a 500-ps period and a 50% duty cycle, corresponding to a sampling rate of 2 GS/s. Half of each clock period (250 ps) is allocated for input signal tracking and sampling, while the remaining 250 ps is used for DAC subtraction and residue amplification. Specifically, during the sampling phase (*ϕ*_1_ high, *ϕ*_2_ low), the input signal is sampled simultaneously onto the capacitor arrays of both the sub-ADC and the MDAC. Simultaneously, the bottom-plate switch *ϕ*_1_*_P_* is turned on to connect the RA input to a common-mode voltage (*V_CM_*), establishing the sampling reference. During the amplification phase (*ϕ*_1_ low, *ϕ*_2_ high), the capacitor arrays switch to the corresponding reference voltages to generate the residue, and the RA operates in a closed-loop amplification mode. To mitigate aperture errors introduced by the SHA-less architecture, the bottom-plate switch (*ϕ*_1_*_P_*) is designed to turn off slightly earlier than the main sampling switch (*ϕ*_1_). Meanwhile, the flash ADC comparator latches at the slightly delayed rising edge of *ϕ*_1_*_P_D_*. This preset time offset (T_delay_) precisely matches the signal propagation delay of the comparator preamplifier, thereby maximizing the timing alignment between the MDAC sampling instant and the flash-ADC decision output, which effectively improves the high-frequency dynamic linearity of the system. Additionally, a pseudo-random bit sequence (PRBS)-controlled dither signal is injected at the RA input to facilitate the background calibration of gain errors. Both the delay of *ϕ*_1_*_P_D_* and the preamplifier offset are adjustable, enabling the background calibration of aperture errors and comparator offsets.

### 2.5. The Designed RA and Output Buffer

When designing the RA, both the gain error and the settling error introduced by the operational amplifier must be considered. The total error, *ε_total_*, can be expressed as:

(2) $$\varepsilon_{total}=\varepsilon_{gain}+\varepsilon_{settling}=\frac{1}{A\beta }+e^{-\omega_{-3\mathrm{dB}}t_{s}}$$

where *A* and *β* are the open-loop gain and feedback factor of the amplifier, respectively, *ω_−_*_3dB_ is the −3 dB bandwidth of the closed-loop amplifier, and *t_s_* is the allocated amplification time. For this 14-bit ADC, the first stage resolves 2.5 effective bits, meaning the subsequent stages must achieve a 12-bit quantization accuracy. To avoid missing codes, the gain error must be strictly constrained to less than 0.5 LSB, yielding:

(3) $$\varepsilon_{gain}=\frac{1}{A\beta }\leq \frac{1}{2^{13}}$$

Since the RA operates in a closed-loop configuration within the ADC, and considering the effects of load capacitance and parasitic capacitance at the amplifier’s input on the feedback factor, the loop gain (*LG*) is chosen as the metric for evaluating gain error. Consequently, the loop gain must satisfy:

(4) $$LG=A\beta \geq 2^{13}=78\mathrm{dB}$$

Assuming the amplifier undergoes a purely exponential settling process, the dynamic settling error must satisfy:

(5) $$\varepsilon_{settling}=e^{-\omega_{-3\mathrm{dB}}t_{s}}<\frac{1}{2^{13}}$$

In this design, with the closed-loop gain set to 4 (corresponding to *β =* 1/4) and an allocated settling time *t_s_* of approximately 125 ps, the required closed-loop bandwidth *f_−_*_3dB_ must satisfy:

(6) $$f_{-3\mathrm{dB}}=\frac{\omega_{-3\mathrm{dB}}}{2\pi }\approx 11.47\mathrm{GHz}$$

High-resolution ADCs require an RA with high linearity and fast, stable settling. Although open-loop amplifiers offer attractive speed and energy efficiency, their gain and linearity are highly sensitive to process, voltage, and temperature (PVT) variations, and wide-input-range operation usually requires complex nonlinear calibration. In deeply scaled 28 nm CMOS, short-channel effects severely degrade intrinsic gain, making it difficult to simultaneously achieve the theoretically required loop gain and a bandwidth beyond 11.47 GHz using gain boosting or multistage cascoding alone [17]. Therefore, this design shifts the emphasis from high open-loop gain to a low-gain, fast-settling strategy, while relying on digital background calibration to recover overall system accuracy. Accordingly, the RA is primarily optimized to satisfy the bandwidth and settling requirements of the system.

**Figure 8:**
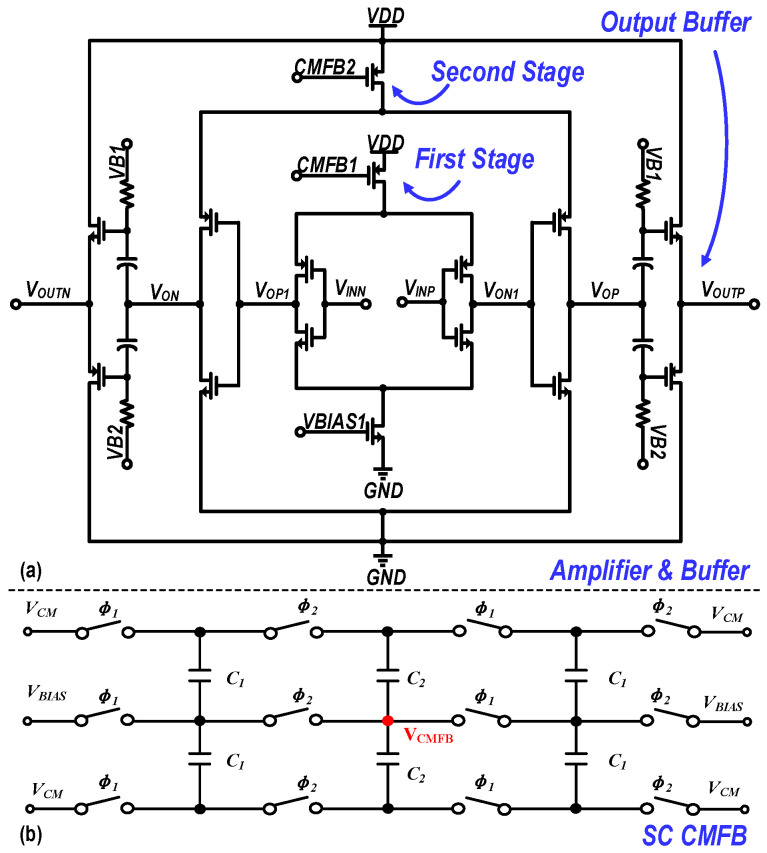
Circuit schematic of the implemented (**a**) RA; (**b**) SC CMFB.

Figure 8a shows the implemented RA and output buffer structure. To meet the requirements for bandwidth, accuracy, and settling speed, a two-stage inverter-based push–pull amplifier is adopted. This is followed by a high-linearity output buffer using AC-coupled biasing to enable rapid settling and linear amplification. In the two-stage RA, both stages exploit the high *g_m_/I_d_* efficiency of the complementary push–pull structure to accelerate settling and improve energy efficiency. Specifically, the first stage utilizes a current-controlled push–pull differential structure, where the tail transistor (*M_N_*_1_) sets the static stage current, and the top transistor (*M_P_*_1_) forms a common-mode feedback (CMFB) loop to regulate the output common-mode level. By contrast, the second stage adopts an unconstrained pseudo-differential push–pull inverter topology. In this second stage, only the top PMOS device provides common-mode regulation without directly limiting the tail current, thereby allowing large transient currents under large-signal operation and effectively improving both slew rate and bandwidth. For output driving, the final buffer employs internal AC-coupling capacitors to excite an independently biased push–pull source follower. This arrangement isolates DC drift from the preceding stage while achieving high energy efficiency, low output impedance, and wideband driving capability.

Figure 8b shows the switched-capacitor (SC) CMFB circuit. In fully differential amplifiers, nonidealities such as device mismatch and parasitic effects may shift the output common-mode level away from its target value; therefore, CMFB is required to stabilize it. Compared with continuous-time CMFB, which reduces output swing and introduces static power consumption, SC CMFB is well suited to the two-phase non-overlapping clock operation of pipelined ADCs, as it preserves output swing without additional static power. As Figure 8b shows, this design adopts a dual-phase SC CMFB. During the sampling phase (*ϕ*_1_), the refreshing capacitor *C*_1_ is charged to capture the desired DC bias. During the redistribution phase (*ϕ*_2_), *C*_1_ is placed in parallel with the continuous-sensing capacitor *C*_2_ to share and redistribute charge, gradually driving the feedback node (*V_CMFB_*) to its steady-state value. An auxiliary set of bias capacitors is introduced to accelerate convergence and ensure effective feedback during both clock phases. The closed-loop accuracy and speed are determined by the gain and bandwidth of the CMFB loop. To balance performance and power, the CMFB loop bandwidth is set to approximately half that of the differential signal path, and proportional replication is used in the bias network to ensure current accuracy. Both RA stages employ SC CMFB to stabilize the output common-mode level across PVT variations.

During the design of the RA, the effects of layout parasitics and PVT variations on the closed-loop response were comprehensively considered. To ensure sufficient design margin, the −3 dB bandwidth of the closed-loop RA is specified to be greater than 12 GHz. By optimizing both the bias-current distribution of the two amplification stages and the device sizing ratios, the design achieves favorable phase stability while satisfying the stringent settling requirement.

**Figure 9:**
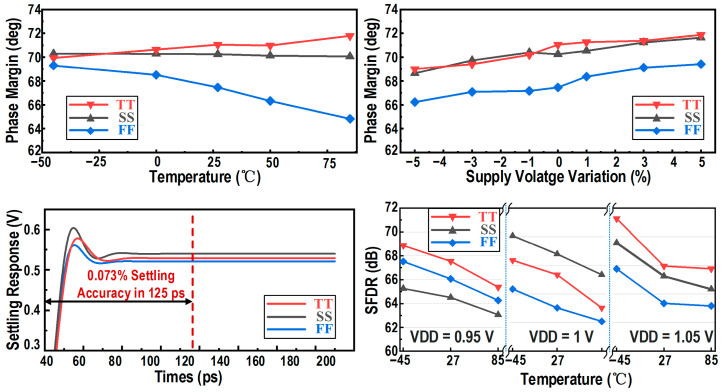
Simulated performance of the designed RA across PVT variations.

Figure 9 presents the comprehensive simulated performance of the RA across various PVT conditions. The top subplots demonstrate that the phase margin consistently exceeds 60° against temperature and supply voltage variations. The bottom-left subplot shows the simulated settling responses under different process corners; even under the worst-case condition, the amplifier reliably settles to the required accuracy within the 125 ps window. Finally, the bottom-right subplot illustrates the simulated output SFDR over a temperature range from −45 °C to 85 °C and under supply-voltage variations of ±5%. As depicted, the designed RA achieves a nominal SFDR of approximately 68–71 dB, and crucially, maintains an SFDR above 62 dB even under the most severe PVT variations, providing a robust analog foundation for the subsequent digital background calibration.

### 2.6. The Designed Comparator with Offset Calibration

As the core unit responsible for analog-to-digital conversion in the flash sub-ADC, the comparator directly determines the upper performance bound of the overall pipelined ADC through key metrics such as decision speed, energy efficiency, and input-referred offset voltage. On the one hand, the feedforward decision delay of the comparator directly encroaches upon the time available for RA settling. On the other hand, since the flash architecture relies on multiple comparators operating in parallel, the associated area and power consumption are also considerable. Furthermore, the input-referred offset of the comparator increases the swing of the DAC-reconstructed residue signal; in severe cases, this may drive the residue beyond the input range of the subsequent stage, causing irreversible nonlinear distortion.

**Figure 10:**
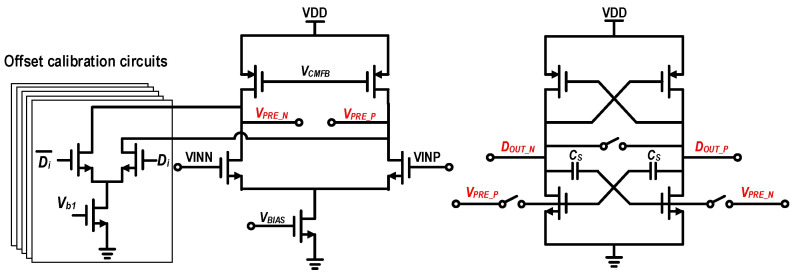
Circuit schematic of the adopted high-speed comparator.

Although conventional fully dynamic comparators offer high speed and zero static power consumption, they suffer from severe kickback noise. This not only degrades the signal integrity at the preceding sampling node but also exacerbates the design complexity of the MDAC. To achieve an optimal trade-off among speed, accuracy, and robustness, this work adopts the high-speed comparator architecture illustrated in Figure 10, which consists of a static preamplifier followed by a dynamic regenerative latch. During the sampling and tracking phase, the differential input signals (*V_INP_* and *V_INN_*) are amplified by the preamplifier. When the latch clock is high, the internal switches are turned on, forcing the differential latch outputs to the same common-mode level and resetting the latch. Meanwhile, the intermediate-node signals (*V_PRE_P_* and *V_PRE_N_*) are continuously tracked and stored at the gates of the latch input transistors. When the latch clock transitions from high to low, the input switches turn off to isolate the preamplifier, and the latch rapidly enters positive-feedback regeneration, converting the amplified differential signal into full-swing digital outputs.

To further accelerate regeneration and shorten the decision time, cross-coupled capacitors (*Cs*) are introduced inside the latch to provide an AC positive-feedback path during the initial regeneration transient. Meanwhile, the preamplifier is equipped with a CMFB circuit to stabilize its output common-mode level and enhance the stability of the DC operating point across PVT variations. In this cascaded architecture, the preamplifier provides fast, low-noise, and linear amplification for small differential inputs, while the latch takes over to complete the large-signal regeneration process. The inclusion of the preamplifier not only boosts the input signal to effectively shorten the total comparison time, but more importantly, its reverse-isolation property greatly suppresses the kickback noise generated during the large-signal regeneration of the latch. Consequently, the linearity of the front-end sampling network is highly preserved.

**Figure 11:**
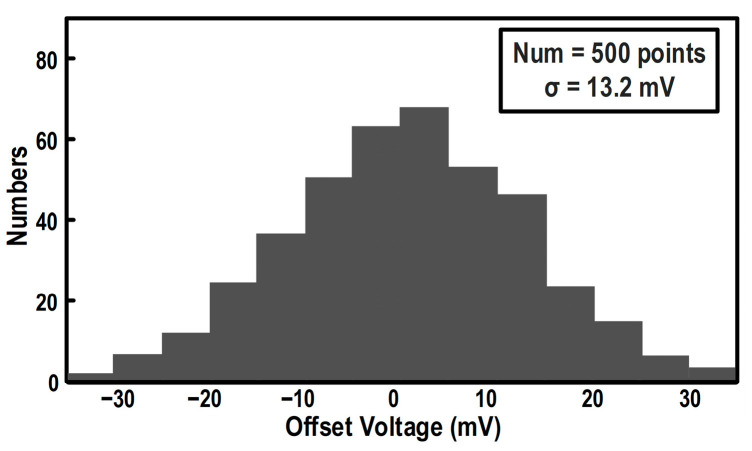
Monte Carlo simulation histogram of the comparator input-referred offset voltage (500 runs).

In this design, to minimize parasitic capacitance and maximize comparison speed, the differential input pair of the preamplifier employs minimum-channel-length devices, which inevitably introduces a non-negligible input offset. Figure 11 presents the simulated comparator input-offset distribution, revealing a standard deviation (σ) of 13.2 mV. To prevent the increased residue swing and the resulting amplification nonlinearity caused by this offset, offset calibration is indispensable.

**Figure 12:**
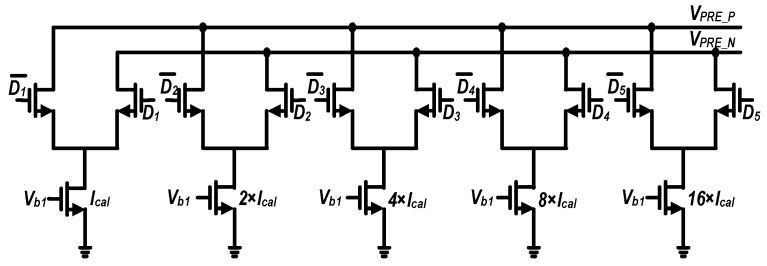
Circuit schematic of the binary-weighted current-source array used for comparator offset calibration.

**Figure 13:**
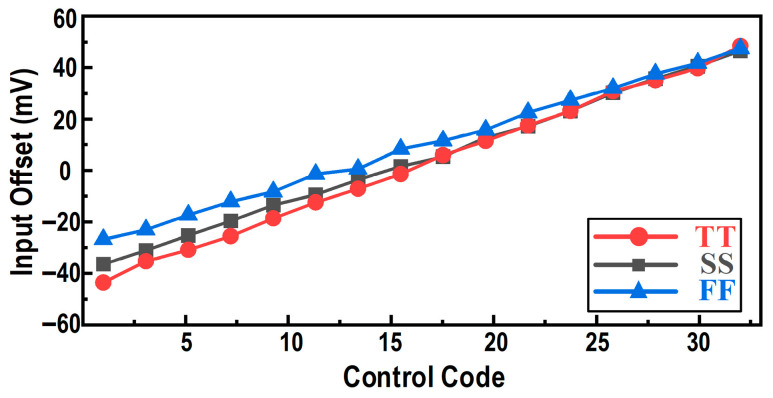
Simulated input offset versus the digital control code across different process corners.

Accordingly, this work adopts the offset-adjustment scheme shown in Figure 12. A digitally controlled current-source array is introduced into the preamplifier to regulate the differential branch currents, thereby enabling precise offset tuning. The current-source array is arranged in a binary-weighted manner to minimize the number of required control switches. Figure 13 shows the simulated relationship between the input offset and the digital control code. The results indicate that, across different process corners, the calibration circuit provides a tuning range exceeding ±40 mV, which fully covers the 3σ variation range (39.6 mV) of the comparator offset.

### 2.7. The CC-VTC

**Figure 14:**
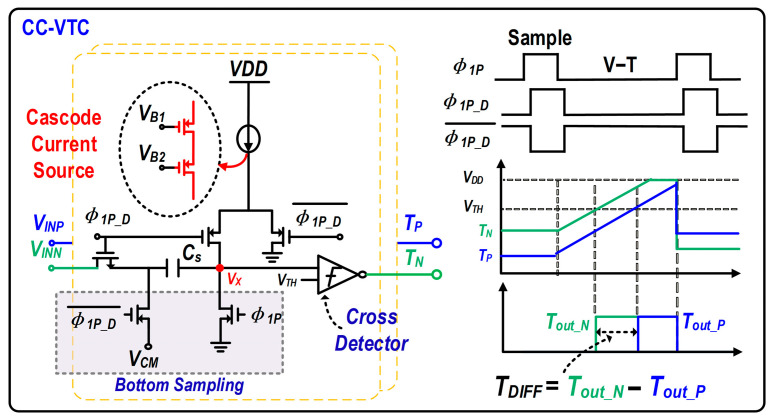
Circuit schematic and timing diagram of the implemented CC-VTC.

Figure 14 presents the circuit topology and timing diagram of the constant-current voltage-to-time converter (CC-VTC) employed in this design. The VTC employs a cascode constant-current source to improve output-current stability, thereby enabling nearly constant-current charging of the capacitor and enhancing the linearity of the voltage-to-time conversion. To reduce the impact of charge injection and clock feedthrough from the sampling switch on conversion accuracy, a bottom-plate sampling technique is adopted.

During operation, when the bottom-plate sampling clock *ϕ*_1_*_P_* is high, the input signal is tracked and sampled onto the sampling capacitor *C_S_*. When *ϕ*_1_*_P_* transitions low, the sampled charge is locked, and the capacitor subsequently begins to be charged by the constant-current source. Once the voltage at node *V_X_* exceeds the threshold voltage (*V_TH_*) of the crossing detector, a rising-edge output pulse (*T_out_N_* or *T_out_P_*) is generated. In this way, an output time difference, *T_DIFF_* = *T_out_N_* − *T_out_P_*, which is approximately linearly proportional to the input differential voltage, can be obtained. In this design, to satisfy the 9-bit resolution requirement of the third stage while meeting the thermal noise specification under a 500 mV_pp_ differential input swing, the required sampling capacitance *C_S_* is calculated to be 25 fF.

### 2.8. The GRO-TDC

**Figure 15:**
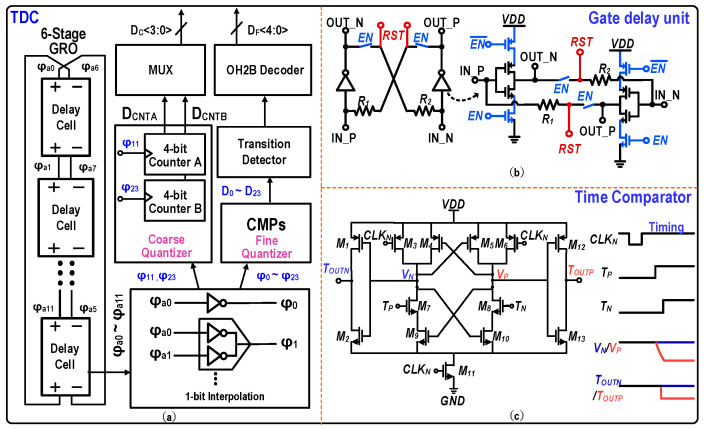
(**a**) Overall architecture of the designed 9-bit GRO-TDC. (**b**) Circuit schematic of the gate delay unit featuring resistive cross-coupled feedforward. (**c**) Circuit schematic and timing diagram of the time comparator.

Figure 15a shows the architecture of the implemented GRO-based TDC. Similar to the structure reported in our previous work [18], the designed 9-bit TDC consists of a 6-stage GRO—where each oscillation cycle generates 12 folded phases—along with a 1-bit interpolation circuit and two 4-bit counters. As depicted in Figure 15b, each delay cell employs a resistive cross-coupled feedforward technique to realize differential delay propagation while reducing the intrinsic propagation delay. Meanwhile, both the gated inverter and the positive-feedback path within each delay cell are tightly controlled by the enable signal (*EN*). The 24 interpolated phases (*ϕ*_0_ *− ϕ*_23_) are resolved by time comparators (CMPs) and sampled by D flip-flops (DFFs). These states are subsequently converted into a 5-bit binary fine output (*D_F_*<*4*:*0*>) through a transition detector and a one-hot-to-binary (OH2B) decoder. To prevent counting errors stemming from the asynchrony between the coarse and fine quantization processes, the coarse quantization is redundantly performed by two independent 4-bit counters triggered by *ϕ*_11_ and *ϕ*_23_, respectively. The final coarse counter output (*D_C_*<*3*:*0*>) is selected by a multiplexer (MUX) according to the transition decision from the fine quantization side. Figure 15c shows the structure of the time-domain comparator employed in this design, which is implemented based on a Strong-ARM latch. When *CLK_N_* is low, the internal nodes (*V_N_* and *V_P_*) are reset to *VDD*, while the input pair is turned off to completely cut off the discharge path. When *CLK_N_* transitions high, the latch enters the comparison state, where the regeneration process is promptly triggered by the earlier-arriving input pulse to complete the decision. Benefiting from the fast signal edges and high-speed switching characteristics inherent in the 28-nm process, this comparator achieves a sub-picosecond time resolution (<1 ps) and a decision delay of less than 50 ps, thereby fully satisfying the stringent requirements of high-speed time-domain comparison.

**Figure 16:**
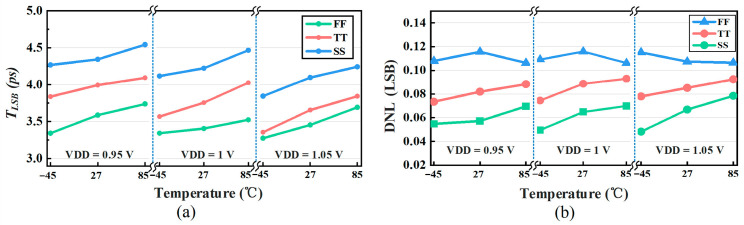
(**a**) Impact of PVT variations in the delay cells on time resolution. (**b**) Impact of PVT variations in the delay cells on linearity.

The GRO in this design operates at 11 GHz, corresponding to an ideal least significant time step (*T_LSB_*) of 3.75 ps. Since the gating delay of the delay cells is sensitive to process, voltage, and temperature (PVT) variations, extensive simulations were carried out across representative PVT corners to evaluate the *T_LSB_* variation and its impact on the quantization linearity. As shown in Figure 16a, under the nominal condition (TT corner, ΔVDD = 0 mV, and 27 °C), the *T_LSB_* is 3.75 ps. Across all simulated PVT conditions, the *T_LSB_* variation reaches up to 20%, which is non-negligible from a system perspective. However, the resulting system-level impact can be substantially mitigated by the proposed background calibration. Furthermore, since all quantization intervals shift in a largely uniform manner under PVT variations, the degradation in quantization linearity remains limited. This is confirmed by Figure 16b, where the maximum DNL remains below 0.12 LSB over all simulated PVT conditions, demonstrating the strong PVT robustness of the proposed quantization architecture. Regarding phase-noise sensitivity, the phase noise of the ring oscillator is translated into time-domain quantization jitter. By employing the resistive cross-coupled feedforward technique in the delay cells, the signal transition edges are sharpened, which helps reduce the phase-noise contribution of individual delay stages and keep the accumulated jitter within the system noise budget.

## 3. Proposed Background Calibration Scheme

To mitigate the nonidealities in the proposed architecture, background calibration is performed sequentially. First, the inter-channel offset and gain mismatches among the four time-interleaved channels in the third stage are calibrated, followed by the timing mismatch calibration. Then, the interstage gain errors are calibrated from the back end to the front end, that is, first between the second and third stages and then between the first and second stages.

### 3.1. Inter-Channel Offset and Gain-Mismatch Calibration

**Figure 17:**
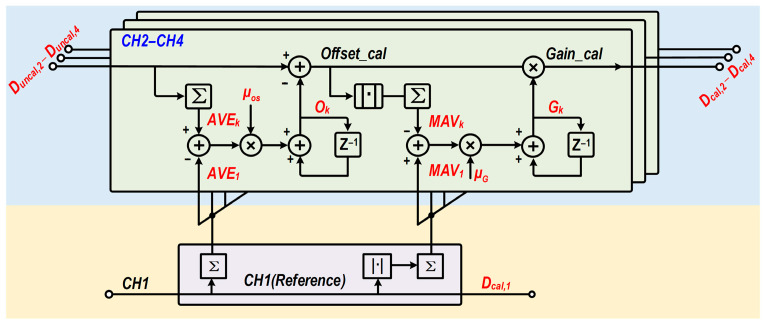
Block diagram of the adopted digital background calibration loop for inter-channel offset and gain mismatches.

In this design, a digital background calibration algorithm based on mean-value and MAV statistics is employed to perform real-time correction of the inter-channel offset and gain mismatches in the four-channel time-interleaved structure. The corresponding offset and gain compensation coefficients are denoted by *O_k_* and *G_k_* for *k* = 2–4, respectively. The block diagram of the adopted calibration loop is illustrated in Figure 17.

During calibration, Channel 1 (*CH1*) is designated as the reference channel, while the remaining channels (*CH2*–*CH4*) are individually tracked and calibrated for offset mismatch and gain error with respect to *CH1*. Inspired by the hardware-efficient algorithms reported in [19], the proposed background calibration loop strictly avoids the use of power-hungry digital multipliers in the error-detection path. By leveraging the mean absolute value (MAV) instead of the mean square value for gain estimation, the digital back-end footprint is significantly minimized while robustly tracking the inter-channel mismatches inherent to the 28-nm process. Specifically, the quantized outputs of all channels are first accumulated and averaged to obtain their mean values, denoted by *AVE_k_* (*k* = 2~4). Subsequently, after removing the offset component, the resulting signal is passed through an absolute-value operation and then accumulated to yield the statistical quantity *MAV_k_* (*k* = 2~4), which reflects the gain magnitude of each channel. Based on these statistical measures, the relative offset error and relative gain error of each channel with respect to *CH1* are defined as:

(7) $$\begin{array}{l} E_{os,k}=AVE_{K}-AVE_{1}\\ E_{G,k}=MAV_{k}-MAV_{1} \end{array}$$

where *E_os,k_* denotes the channel offset mismatch error of the *k*-th channel relative to the reference channel, and *E_G,k_* denotes the corresponding gain error. On this basis, the least mean squares (LMS) adaptive algorithm is employed to iteratively update the offset compensation coefficient *O_k_* and the gain compensation coefficient *G_k_* for each channel. To establish a stable negative-feedback closed loop, the iterative expressions are given by:

(8) $$\begin{array}{l} O_{k}[n+1]=O_{k}[n]+\mu_{os}\times E_{os,k}[n]\\ G_{k}[n+1]=G_{k}[n]+\mu_{G}\times E_{G,k}[n] \end{array}$$

where *μ_os_* and *μ_G_* represent the convergence step sizes for offset calibration and gain calibration, respectively. Through the foregoing closed-loop iterations, the loop adaptively converges to a steady state. During calibration, the raw output *D_uncal,k_* is first corrected by subtracting the offset compensation term, and is then normalized by the gain compensation coefficient. Therefore, the final calibrated quantized output of the *k*-th channel can be expressed as:

(9) $$D_{cal,k}=(D_{uncal,k}-O_{k})/G_{k}$$

In this way, the channel offset mismatch and gain errors among the third-stage interleaved channels are calibrated.

**Figure 18:**
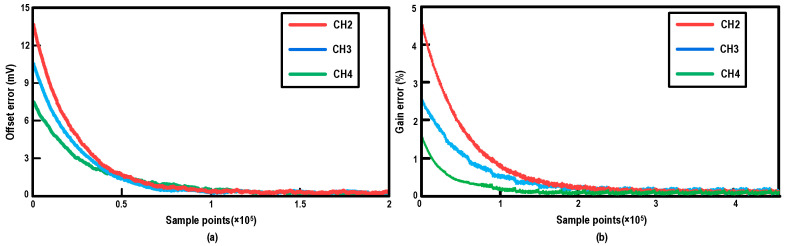
Simulated convergence trajectories of the digital background calibration. (**a**) Inter-channel offset error versus sample points. (**b**) Inter-channel gain error versus sample points.

To verify the dynamic characteristics and robustness of the proposed algorithm, the convergence behavior is quantitatively evaluated. Figure 18 plots the simulated offset and gain error tracking trajectories versus the number of sample points for the interleaved channels (CH2, CH3, and CH4) relative to the reference channel (CH1). As shown in Figure 18a, the initial offset errors are smoothly compensated, converging stably to near zero after approximately 1.5 × 10^7^ sample points. Similarly, Figure 18b demonstrates that the inter-channel gain errors adaptively settle to their steady states within 2.5 × 10^7^ sample points. These convergence results demonstrate the stable operation of the proposed MAV-based negative-feedback loop. They indicate that the proposed scheme can effectively track and mitigate process-induced mismatches without requiring digital multipliers in the error-detection path. In this way, the channel offset and gain mismatches among the third-stage interleaved channels are calibrated.

### 3.2. Time Mismatch Calibration

**Figure 19:**
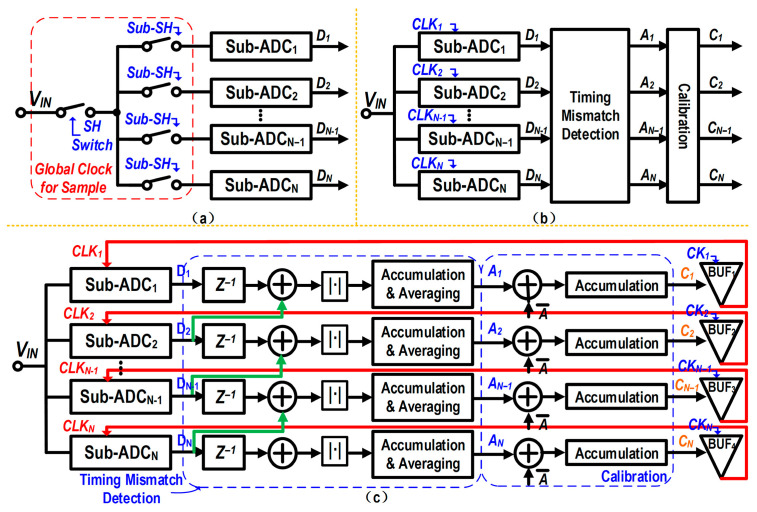
Timing mismatch calibration strategies: (**a**) Conventional unified front-end sampling. (**b**) Distributed mixed-signal calibration architecture. (**c**) Block diagram of the adopted MAV-based digital background detection and analog delay control loop.

A commonly used approach for timing-mismatch calibration is the unified front-end sampling scheme, as shown in Figure 19a. By employing a shared front-end master sampler controlled by a global clock, the sampling instant of each channel can be uniformly defined, thereby eliminating timing mismatches at the physical level [20]. However, once the sampling rate exceeds 1 GS/s, the drive capability and high-frequency bandwidth of the large front-end sampling switch often become the system bottleneck due to CMOS technology limitations. Accordingly, this design adopts a mixed-signal calibration strategy introduced in [21], which is based on the mean absolute value (MAV) of the output differences between adjacent channels. The algorithm compensates for timing mismatches by extracting the timing error in the digital background and feeding this error signal back to the analog domain to adjust the digitally controlled delay lines (DTCs). This method avoids the use of complex multipliers and finite impulse response (FIR) filters, thereby substantially reducing the computational burden on the digital baseband. From the perspective of analog implementation, it merely requires inserting these low-power DTCs into the sampling-clock path of each sub-ADC channel. As shown in Figure 19b, this specific modification does not alter the principal analog architecture of the individual sub-channels. The specific procedure for the digital detection and feedback control, implemented based on the method in [21], is illustrated in Figure 19c.

First, the difference Δ*D_k_*[*m*] between the quantized outputs of adjacent channels is computed, where *m* denotes the clock-cycle index in parallel processing. Taking into account the cyclic nature of the *N*-channel time interleaving, the difference extraction can be expressed as:

(10) $$\Delta D_{k}[m]=\left\{\begin{array}{l} D_{k}[m]-D_{k-1}[m],k=2,3,\ldots ,N\\ D_{1}[m]-D_{N}[m-1],k=1 \end{array}\right.$$

Subsequently, the absolute value of this difference sequence is taken. Accumulation and averaging are then performed over a defined time window with length *M* to obtain the statistical mean of the absolute differences for each channel, denoted by *A_k_*:

(11) $$A_{k}=\frac{1}{M}\sum_{m=1}^{M}\left|\Delta D_{k}[m]\right|$$

Provided that the input signal possesses sufficient statistical richness, all channel-specific mean values *A_k_* will theoretically be identical in the absence of timing mismatches, since the sampling intervals among the channels are exactly equal. Conversely, if a given channel samples either earlier or later than intended, the temporal spacing between adjacent sampling points is altered, causing the corresponding *A_k_* to increase or decrease accordingly. To extract a purely timing-related error signal, the system further computes the global average of the mean values across all *N* channels:

(12) $$\bar{A}=\frac{1}{N}\sum_{k=1}^{N}A_{k}$$

On this basis, the relative timing-deviation indicator for the *k*-th channel, *E_t,k_* can be extracted by comparing *A_k_* with the global average $\bar{A}$, as follows:

(13) $$E_{t,k}=A_{k}-\bar{A}$$

The resulting error signal is then integrated by a digital accumulator to generate the digital calibration code *C_k_* that controls the analog adjustable delay buffer:

(14) $$C_{k}[n+1]=C_{k}[n]-\mu_{t}\cdot E_{t,k}$$

Here, *μ_t_* denotes the convergence step size of the timing-calibration loop, and *n* is the iteration index. This calibration code *C_k_* is fed back to the delay buffer of the corresponding channel, thereby dynamically adjusting the absolute phase of the sub-ADC sampling clock *CLK_k_*. As the feedback loop iterates continuously, the values of *A_k_* across all channels gradually converge to $\bar{A}$, and the error signal *E_t,k_* approaches zero. In this way, the timing mismatch among the interleaved channels is effectively eliminated at the analog physical level, substantially suppressing interleaving spurs and enhancing overall linearity.

**Figure 20:**
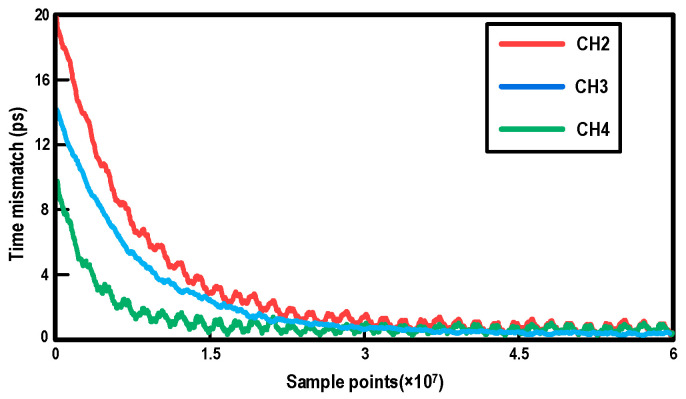
Simulated convergence trajectories of the timing mismatches versus sample points for the interleaved channels.

To quantitatively evaluate the effectiveness and dynamic characteristics of the timing mismatch calibration, system-level simulations were performed. Figure 20 illustrates the simulated convergence trajectories of the timing mismatches for the interleaved channels (CH2, CH3, and CH4) relative to the sampling instant of CH1 versus the number of sample points. Driven by the mixed-signal feedback loop, the initial timing mismatches are continuously corrected and smoothly settle to near zero. As depicted in the figure, the calibration loop achieves steady-state convergence after approximately 4.0 × 10^7^ sample points, successfully overcoming significant initial deviations. The slight residual ripples at the steady state indicate the dynamic tracking nature of the loop and remain small in the simulation results. This convergence behavior demonstrates the robustness of the adopted MAV-based algorithm in effectively mitigating timing mismatches while avoiding complex digital multipliers.

### 3.3. Interstage Gain-Mismatch Calibration

After completing the calibration of channel offset mismatches and gain errors for the four interleaved channels in the third stage, the interstage gain error between the second and third stages is calibrated. The calibrated quantized outputs of all third-stage channels are combined and denoted as *D*_3_*_cal_*. Subsequently, an LMS adaptive algorithm based on pseudo-random dither injection is employed to iteratively compute the interstage gain weight (*G*_2_) by correlating *D*_3_*_cal_* with the injected dither (*G_dither_*_2_). The calibration update expression is as follows:

(15) $$G_{2}[n+1]=G_{2}[n]+\mu_{2}\cdot G_{dither2}[n]\cdot (D_{3\_cal}[n]-G_{dither2}[n]\cdot G_{2}[n])$$

where *μ*_2_ is the convergence step size. After interstage gain-error calibration is completed and the digital equivalent of the injected dither (*G_dither_*_2_) is subtracted, the combined output of the second and third stages, denoted as *D*_2_*_cal_*, can be expressed as:

(16) $$D_{2cal}[n]=G_{2}[n]\cdot D_{2}[n]+D_{3cal}[n]-G_{2}[n]\cdot G_{dither2}[n]$$

Subsequently, the interstage gain error between the first and second stages (*G*_1_) is calibrated using the first-stage injected dither (*G_dither_*_1_) and the LMS algorithm, as follows:

(17) $$G_{1}[n+1]=G_{1}[n]+\mu_{1}\cdot G_{dither1}[n]\cdot (D_{2cal}[n]-G_{dither1}[n]\cdot G_{1}[n])$$

Finally, after calibrating the interstage gain error between the first stage and the subsequent stages and subtracting *G_dither_*_1_, the final combined output of all three pipeline stages, denoted as *D_outcal_*, can be expressed as:

(18) $$D_{out\_cal}[n]=G_{1}[n]\cdot D_{1}[n]+D_{2cal}[n]-G_{1}[n]\cdot G_{dither1}[n]$$

**Figure 21:**
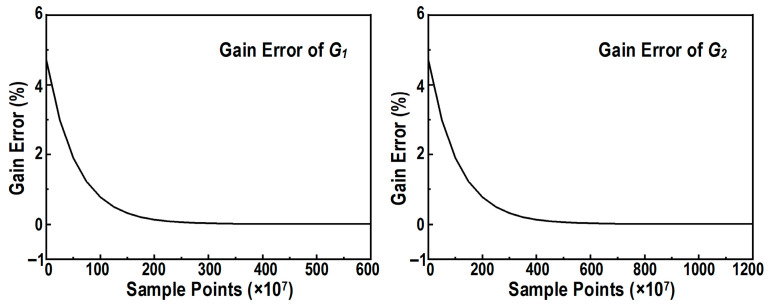
Simulated convergence trajectories of the interstage gain errors (*G*_1_ and *G*_2_) using the implemented LMS-based digital background calibration.

In this manner, the digital background calibration of all interstage gain errors is fully accomplished. Figure 21 illustrates the convergence trajectories of the interstage gain errors between the first and the second stages (*G*_1_), and between the second and the third stage (*G*_2_) respectively. Simulation results indicate that, after robust convergence, the residual gain error of *G*_1_ is reduced to less than 0.032%, while that of *G*_2_ is minimized to less than 0.085%.

**Figure 22:**
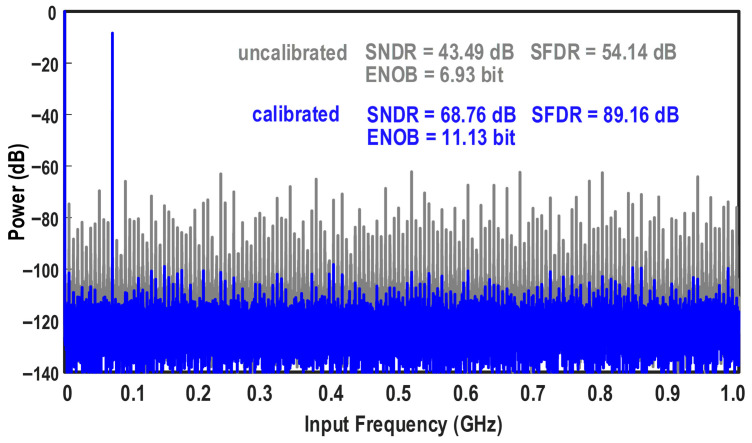
Simulated FFT spectra of the ADC before and after the digital background calibration.

To verify the implemented calibration algorithm, a model of this ADC was constructed in MATLAB R2024a, and non-idealities were artificially introduced. Figure 22 presents the simulated FFT spectra of the ADC before and after calibration. Following the application of the digital background calibration, the harmonic and spurious powers are effectively suppressed. The dynamic performance of the ADC is significantly improved: the SNDR increases by 25.27 dB, from 43.49 dB to 68.76 dB, and the SFDR increases by 35.02 dB, from 54.14 dB to 89.16 dB. Correspondingly, the ENOB improves from 6.93 bits to 11.13 bits. These simulation results demonstrate that the adopted digital background calibration effectively enhances the overall linearity and dynamic performance of the ADC.

## 4. Measurement Results

**Figure 23:**
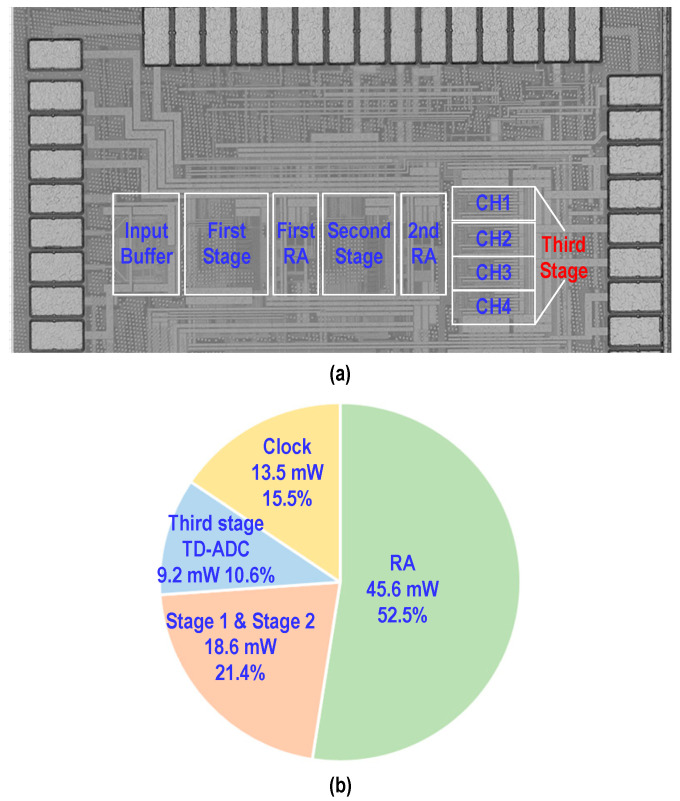
(**a**) Layout of the prototype ADC in 28-nm CMOS. (**b**) Power breakdown of the ADC core.

Figure 23a shows the layout of the prototype chip implemented in this work, which is fabricated in a 28-nm CMOS process and occupies an active core area of 0.16 mm^2^.

The corresponding power breakdown is illustrated in Figure 23b. Excluding the input buffer and the reference-voltage driver, the ADC core consumes 86.9 mW. Due to the stringent bandwidth requirement imposed on the amplifier, the RAs account for the largest portion of power dissipation, approximately 45.6 mW. Under a 1-V supply voltage, the first- and second-stage pipelined structures together consume 18.6 mW, while, benefiting from the high energy efficiency of time-domain quantization, the third stage consumes only 9.2 mW. In addition, the clock distribution circuitry dissipates approximately 13.5 mW.

**Figure 24:**
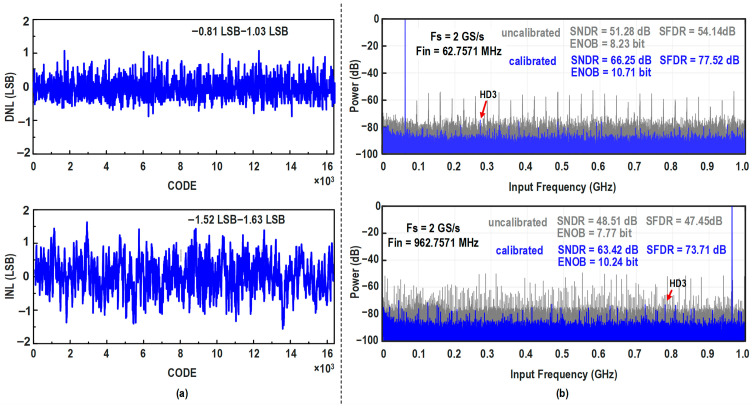
Measured dynamic and static performance of the prototype ADC at 2 GS/s: (**a**) DNL and INL. (**b**) FFT spectra for low-frequency (62.76 MHz) and near-Nyquist (962.76 MHz) inputs.

**Figure 25:**
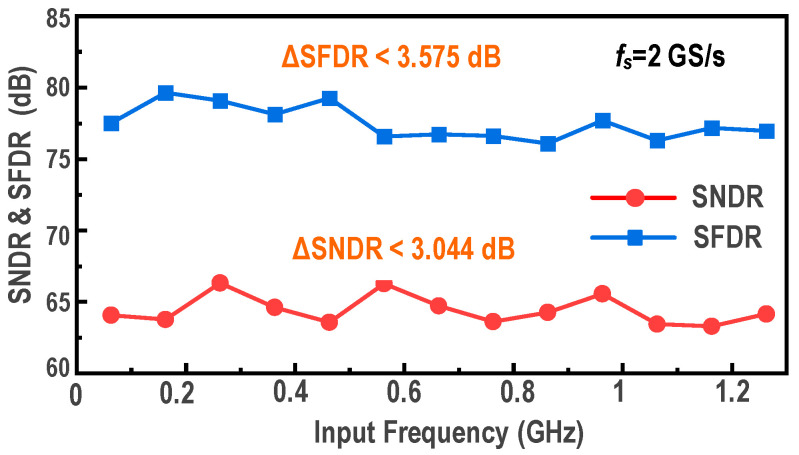
Measured dynamic performance (SFDR and SNDR) versus input frequency up to 1.2 GHz at a 2-GS/s sampling rate.

The measured DNL and INL of the calibrated ADC are shown in Figure 24a. The DNL ranges from −0.81 LSB to +1.03 LSB, while the INL varies from −1.52 LSB to +1.63 LSB. To demonstrate the effectiveness of the background calibration, the measured FFT spectra before and after calibration at a sampling rate of 2 GS/s are presented in Figure 24b. When the input frequency is approximately 62.76 MHz, the uncalibrated SNDR and SFDR are limited to 51.28 dB and 54.14 dB, respectively. After the calibration, the measured SNDR and SFDR increase to 66.25 dB and 77.52 dB, respectively, corresponding to an ENOB of 10.71 bits. Similarly, at a near-Nyquist input frequency of 962.76 MHz, the uncalibrated SNDR and SFDR drop to 48.51 dB and 47.45 dB, respectively. With the background calibration activated, the measured SNDR and SFDR are restored to 63.42 dB and 73.71 dB, maintaining an ENOB of 10.24 bits. The measured SNDR and SFDR versus input frequency are shown in Figure 25. Across the input-frequency range from low frequency to 1.2 GHz, the ADC maintains robust dynamic linearity, with an SNDR exceeding 62 dB, an SFDR exceeding 70 dB, and an ENOB above 10.0 bits.

**Figure 26:**
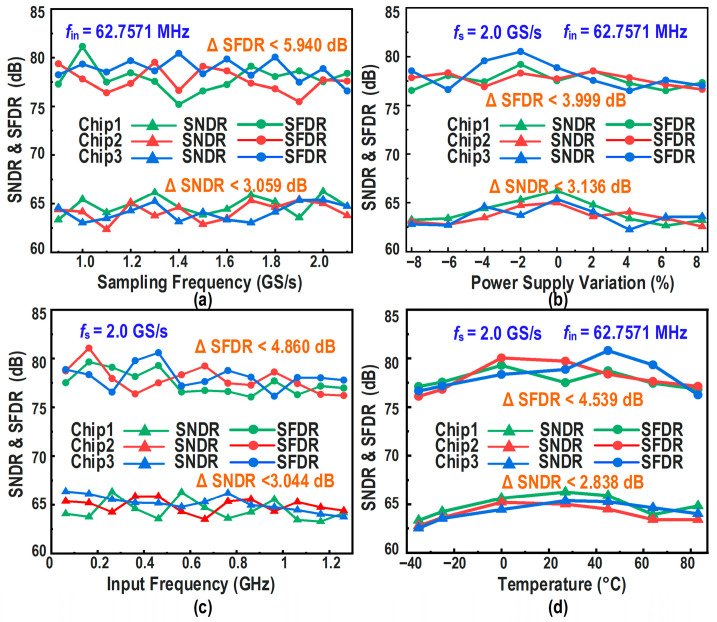
Measured robustness of dynamic performance across three prototype chips versus: (**a**) sampling frequency, (**b**) power supply variation, (**c**) input frequency, and (**d**) temperature.

To further verify the process consistency and environmental robustness of the implemented architecture, three prototype chips were tested under various operating conditions, and the measured dynamic performance variations are presented in Figure 26. Figure 26a shows the measured SNDR and SFDR of the three chips as functions of sampling rate (*f_s_*) at room temperature (27 °C), with an input frequency (*f_in_*) of 62.75 MHz. The results show that, over the sampling-rate range up to 2.1 GS/s, all three chips achieve an SNDR greater than 62 dB and an SFDR greater than 75 dB, with the SNDR variation confined to less than 3.059 dB and the SFDR variation confined to less than 5.940 dB. Figure 26b presents the measured dynamic performance as a function of power supply variation from −8% to +8% under a 2.0-GS/s sampling rate. The results show that, over the supply-voltage variation range of ±8%, all three chips achieve an SNDR greater than 62 dB and an SFDR greater than 76 dB, with the SNDR variation confined to less than 3.136 dB and the SFDR variation confined to less than 3.999 dB. Figure 26c shows the frequency-sweep results of SNDR and SFDR versus input frequency up to 1.2 GHz at 2.0 GS/s. The results show that, over the input-frequency range up to 1.2 GHz, all three chips achieve an SNDR greater than 63 dB and an SFDR greater than 76 dB, with the SNDR variation confined to less than 3.044 dB and the SFDR variation confined to less than 4.860 dB. Finally, Figure 26d plots the measured SNDR and SFDR versus temperature under the same sampling-rate and low-frequency input conditions. The results show that, over the temperature range from −45 °C to 85 °C, all three chips achieve an SNDR greater than 62 dB and an SFDR greater than 76 dB, with the SNDR variation confined to less than 2.838 dB and the SFDR variation confined to less than 4.539 dB. Taken together, these measurements demonstrate that the proposed ADC exhibits good inter-die consistency and robust operation across variations in sampling rate, input frequency, supply voltage, and temperature.

Table 1 summarizes the performance of the prototype ADC and compares it with state-of-the-art ADCs with similar specifications. Benefiting from the highly efficient time-domain quantization and the comprehensive digital background calibration technique, this design achieves competitive dynamic performance at the Nyquist frequency, recording an SNDR of 63.42 dB and an SFDR of 73.71 dB (yielding an ENOB of 10.24 bits). Furthermore, the core power consumption is only 86.9 mW at 2.0 GS/s, translating to a Walden FoM of 35.9 fJ/conv.-step. This demonstrates that the proposed hybrid-domain architecture offers a power-efficient solution with competitive performance relative to recently reported ADCs of similar specifications.

## 5. Conclusions

This paper has presented a 2-GS/s voltage–time hybrid pipelined ADC with a 14-bit digital output in a 28-nm CMOS process. By combining voltage-domain coarse quantization with time-domain fine quantization, the proposed architecture provides an effective solution for balancing resolution, speed, and power efficiency in deeply scaled technologies. Compared with previously reported hybrid voltage–time ADCs in [12,15], this work extends the hybrid concept from medium-resolution designs to a high-resolution GS/s-class pipelined ADC through a co-designed three-stage conversion chain. In addition, unlike the hybrid pipelined ADC reported in [16], the proposed multiplier-less digital background calibration framework is able to simultaneously compensate for inter-channel timing, offset, and gain mismatches, as well as interstage gain errors, thereby improving overall linearity with low hardware overhead. Circuit-wise, the proposed ADC incorporates a SHA-less front-end for wideband sampling, a low-gain inverter-based push–pull residue amplifier for energy-efficient large-signal settling, a highly linear CC-VTC for voltage-to-time conversion, and a four-channel time-interleaved GRO-TDC for fine quantization. Measurement results show that the prototype achieves an SNDR of 66.25 dB at 62.76 MHz and maintains an SNDR of 63.42 dB and an SFDR of 73.71 dB at a near-Nyquist input frequency of 962.76 MHz. Excluding the input buffer and the reference-voltage driver, the ADC core consumes 86.9 mW from a 1-V supply and achieves a Walden FoM of 35.9 fJ/conv.-step. These results demonstrate that the proposed architecture offers a power-efficient and calibration-friendly solution for high-speed, high-resolution ADC design in advanced CMOS processes. Future work will investigate further optimization of the hybrid-domain architecture in more advanced technology nodes to extend its performance toward next-generation ultra-wideband applications.

## Figures and Tables

**Table 1:** Performance Summary and Comparison with State-of-the-Art High-Speed ADCs.

	[22]	[23]	[24]	[25]	[26]	This Work
Architecture	Pipeline	TI Pipeline	Pipeline-SAR	Pipeline	TI Pipeline-SAR	Hybrid Pipeline
Technology (nm)	28	65	16	28	28	28
Resolution (bits)	14	12	13	12	13	14
Sampling rate (GS/s)	2.5	1	4	1	2	2
SNDR@Nyq. (dB)	62	56.2	57.3	61.2	60.36	63.42
SFDR@Nyq. (dB)	73	71.35	67.0	83.2	71.86	73.71
ENOB (bits)	10.01	9.04	9.23	9.87	9.73	10.24
DNL (LSB)	-	-	−0.3 to +0.3	−0.6 to +0.6	−0.39 to +0.29	−0.81 to +1.03
INL (LSB)	-	-	−1.5 to +1.5	−1.0 to +0.8	−1.73 to +2.29	−1.52 to +1.63
Power (mW)	1150 ^1^	31.5 ^2^	513	36.5	252.6	86.9 ^2^
FoMw (fJ/conv.-step)	447.2 ^1^	59.7 ^2^	214.2	39.3	148.3	35.9 ^2^
Core Area (mm^2^)	14.4 ^1^	0.27 ^2^	1.04	0.068 ^1^	1.062	0.16 ^3^

^1^ Full-chip power and area. ^2^ ADC-core power, excludes input buffer and reference driver. ^3^ ADC-core area including input buffer.

## Data Availability

The original contributions presented in this study are included in the article. Further inquiries can be directed to the corresponding author.
